# Anesthetic management with subcostal transversus abdominis plane block in recessive dystrophic epidermolysis bullosa for peritoneal dialysis catheter replacement: a case report

**DOI:** 10.1186/s40981-018-0175-0

**Published:** 2018-05-08

**Authors:** Katsuhiro Aikawa, Nobuhiro Tanaka, Yuji Morimoto

**Affiliations:** Department of Anesthesiology and Critical Care Medicine, Faculty of Medicine and Graduate School of Medicine Hokkaido University, Kita-15, Nishi-7, Kita-ku, Sapporo, 060-8638 Japan

**Keywords:** Recessive dystrophic epidermolysis bullosa, Subcostal transversus abdominis plane block, Placement of peritoneal dialysis catheter

## Abstract

**Background:**

Recessive dystrophic epidermolysis bullosa (RDEB) is a rare, hereditary mucocutaneous disorder that can involve renal insufficiency. If a vascular access for hemodialysis is unavailable, peritoneal dialysis can be utilized. This report describes an anesthetic management with ultrasound-guided transversus abdominis plane block (TAPB) in a patient with RDEB for peritoneal dialysis catheter replacement.

**Case presentation:**

A 49-year-old woman with RDEB needed to undergo peritoneal dialysis catheter replacement. As general, neuraxial and local infiltration anesthesia can lead to serious complications; we planned anesthetic management with subcostal TAPB as the primary analgesia modality. In the operating theater, surgery was initiated after performing left-sided subcostal TAPB. The patient complained of moderate pain at some points during surgery, and the pain was controlled with intravenous or local anesthetics without serious complications.

**Conclusions:**

In summary, subcostal TAPB could be a useful option for peritoneal dialysis catheter surgery in patients with RDEB.

## Background

Epidermolysis bullosa is a hereditary cutaneous disorder characterized by extreme vulnerability of the skin. Particular caution, known as the “no touch principal,” is required in the anesthetic management of patients with epidermolysis bullosa [1]. Among the various subtypes of epidermolysis bullosa, recessive dystrophic epidermolysis bullosa (RDEB) is the severest form involving mucosal membrane and systemic diseases such as renal insufficiency as well as abnormal skin fragility [2].

In some cases, RDEB involves renal insufficiency requiring dialysis therapy. If vascular access cannot be used due to the patient’s condition, peritoneal dialysis can be utilized as an alternative [3]. Peritoneal dialysis catheter placement is performed under general or neuraxial or local anesthesia [4]; however, all of these techniques can cause serious complications in patients with RDEB. General anesthesia bears a high risk because airway manipulation can cause mucosal blister formation in the upper airway following critical airway obstruction. Furthermore, potential difficult airway may prevent gentle airway manipulation and increase the risk [5, 6]. Neuraxial anesthesia should be avoided if the skin lesions at the potential site of puncture [2]. Moreover, a large dose of subcutaneous local anesthesia can lead to new skin lesions [7].

In recent years, there have been some reports of care with ultrasound-guided transversus abdominis plane block (TAPB) for the placement of a peritoneal dialysis catheter [4, 8]. Moreover, utilizing ultrasound-guided regional anesthesia in patients with RDEB has also been reported [5, 6]. In this case report, we describe the anesthetic management with ultrasound-guided subcostal TAPB for the replacement of a peritoneal dialysis catheter in a patient with RDEB. Written permission to compose this case report was provided by the patient.

## Case presentation

A 49-year-old woman (152 cm, 33.5 kg, blood albumin concentration 1.9 g/dL) who had received peritoneal dialysis needed to undergo dialysis catheter replacement. She had been diagnosed with RDEB in childhood and developed IgA nephropathy as well as severe mucocutaneous disorders. She had begun receiving hemodialysis via central venous catheterization 7 years previously; however, her central veins had been damaged because of repeated catheterizations. Consequently, conversion to peritoneal dialysis had become necessary 4 years before. Although the initial peritoneal dialysis catheter placement had been performed under local anesthesia by surgeons, this technique should be limited because there is potential risk of accidental injection of local anesthesia between the lamina densa and the epidermis following severe skin lesioning [5]. Moreover, alternative forms of anesthesia need to be considered because of the patient’s experience of unbearable pain.

Preoperative evaluation revealed Mallampati class IV, limited mouth opening, and severe adhesion in the oral cavity. Considering the potential upper airway blistering following critical airway obstruction as well as difficult airway, general anesthesia was deemed problematic. Neuraxial anesthesia was precluded because of an existing skin lesion at the potential puncture site. After careful consideration, we planned anesthetic management with ultrasound-guided subcostal TAPB as the primary analgesia modality, based on previous reports [4, 8].

In the operating theater, after carefully fitting a pulse oximeter and blood pressure cuff, as performed in previous studies [1, 9], a single shot of fentanyl 25 μg, ketamine 5 mg, and propofol (target-controlled infusion 1.0–1.5 μg/mL) was administered. Subsequently, left-sided ultrasound-guided subcostal TAPB was performed. After infiltration with a small dose of lidocaine, a 20-gauge, 12-cm needle was inserted via an in-plane technique. After excluding intravascular injection, 20 mL of 0.5% lidocaine combined with epinephrine was injected. In patients with RDEB, particular caution is required to avoid causing new bulla formation through friction caused by the ultrasound probe [6]. To this end, we used sufficient amounts of gel and avoided performing sliding movements for as long as possible to minimize shearing force. Contrary, as vertical force to the skin is relatively tolerable [1], we could obtain a clear ultrasound image using the technique of pressing the probe against the skin.

**Fig. 1:**
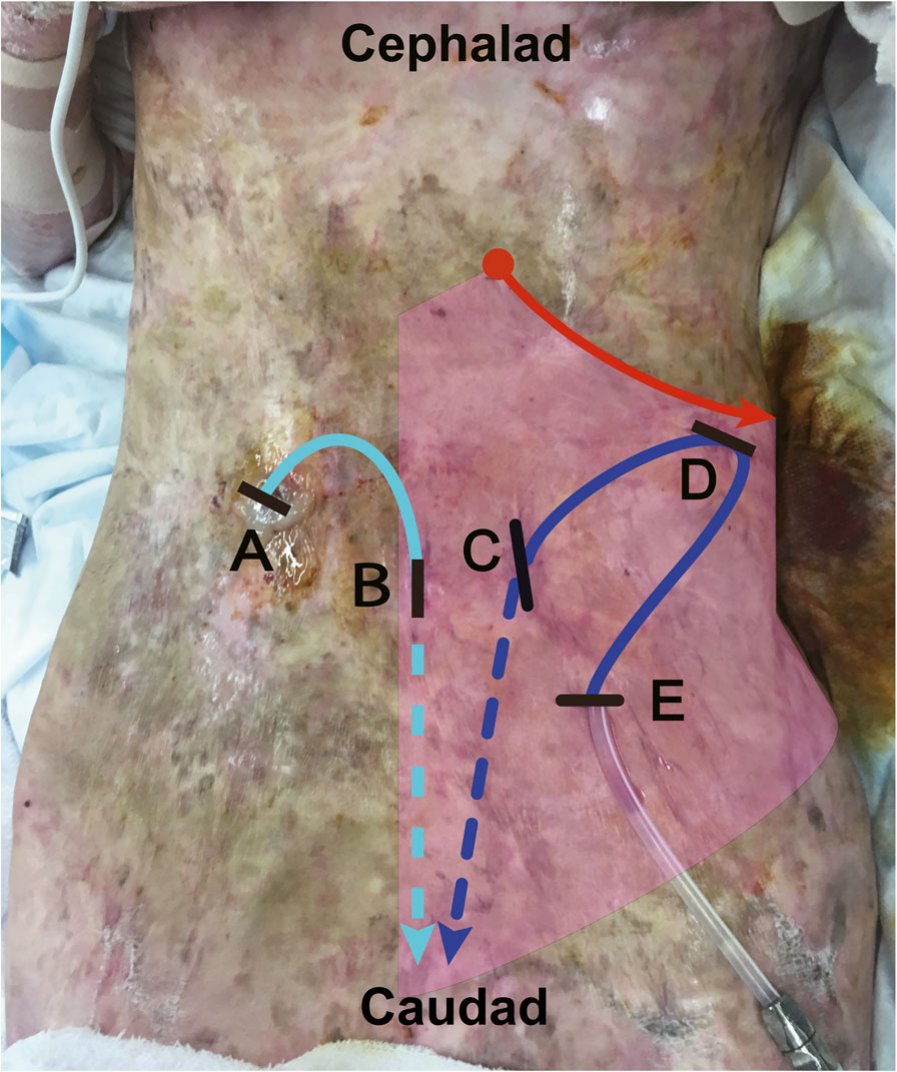
Postoperative image of the surgery site. The incision points and course of removed (light blue arrow) and newly inserted (blue arrow) peritoneal dialysis catheters are shown. The subcutaneous traveling is indicated by solid lines, and the insertion into the peritoneum cavity is indicated by broken lines. The black lines represent incisions (A–E). The red dot and arrow along the subcostal oblique line indicate the injection point and course of local anesthesia via subcostal transversus abdominis plane block. The light-red coloration indicates the expected analgesic area

The surgery was initiated after confirming loss of cold sensation from T7 to T12 in the left anterior abdominal wall. The postoperative image is shown as Fig. 1 to illustrate the incision points and coursing of the catheters. First, the surgeons removed the original catheter. The catheter had been inserted from point A and reached to point B via a subcutaneous tunnel, subsequently penetrating the peritoneal cavity. Although adequate analgesia was provided for point B, the patient complained of moderate pain when point A was incised after local lidocaine infiltration, at the non-blocked side. We managed the pain with additional lidocaine infiltration and intravenous fentanyl and ketamine administration. After removal of the original catheter, the new catheter was placed in her left abdominal wall. Although this side was blocked, she complained of mild pain when the surgeons incised point D, which was controlled with supplemental lidocaine infiltration and intravenous ketamine administration.

The duration of anesthesia was 128 min, and the total dose of anesthetics was composed of fentanyl 200 μg, ketamine 50 mg, and local-infiltration 1% lidocaine 4.4 mL. The patient did not develop serious respiratory depression, unbearable pain, or any serious complication associated with anesthetic management.

## Discussion

Anesthetic management in patients with RDEB is challenging, and an anesthetic plan has to be determined depending on individual conditions [5]. In the present case, general, neuraxial, and large-dose local anesthesia posed potential risks of serious complications. General anesthesia had to be avoided because of the presence of difficult airway and critical airway obstruction. Although neuraxial anesthesia could be applicable in a patient with RDEB [9], we found no intact areas at the potential puncture site.

To the best of our knowledge, this is the first report of anesthetic management with ultrasound-guided subcostal TAPB in a patient with RDEB. TAPB is a technique that can provide efficient somatic analgesia by anesthetizing the anterior branch of the spinal nerve [10], and the use of ultrasound is advantageous [4]. Since patients with RDEB usually have low amounts of fat deposits due to malnutrition, we consider that performing ultrasound-guided subcostal TAPB may be feasible in this patient population.

When using subcostal TAPB for peritoneal dialysis catheter placement, physicians should remember that it is ineffective for visceral pain and does not always cover the area innervated by lateral cutaneous branches [11]. In the current case, the patient complained of mild pain when the surgeons incised her lateral abdominal wall on the blocked side (Fig. 1, incision D). To manage visceral pain due to manipulation of the peritoneum, we administered repeated low doses of fentanyl, careful not to cause respiratory suppression. The patient did not complain of visceral pain during the manipulation of the peritoneum. We consider that visceral pain during peritoneal catheter surgery can be controlled with low doses of fentanyl [4].

Although she reported in the postoperative interview that she experienced considerably less pain during this procedure than during the initial catheter placement, we consider that the moderate pain associated with incision A (Fig. 1) should have been avoided. Li et al. [12] reported superior analgesia and patient satisfaction with TAPB than with local anesthesia during the placement of a peritoneal dialysis catheter. It is possible that bilateral subcostal TAPB would have improved the quality of analgesia in the present case. However, we avoided bilateral TAPB because it would require a near maximum dose of lidocaine combined with epinephrine. As Griffiths et al. [13] reported, TAPB is associated with high risk of local anesthetic systemic toxicity (LAST). Moreover, as Yamamoto et al. reported [4], management of peritoneal dialysis catheter surgery with TAPB may require additional local anesthesia intraoperatively. Therefore, we chose to perform TAPB to the left side, which was exposed to more invasive manipulation, to allow surgeons to utilize additional local anesthesia intraoperatively. Although we believe that the patient’s pain was almost entirely controlled with additional local anesthesia, ketamine, and fentanyl, considering the course of the original catheter, right-sided rectus sheath block could have provided better analgesia. This technique may be safer than TAPB because it requires a smaller dose of local anesthetic and is associated with lower plasma concentration after the procedure [14].

We selected 0.5% lidocaine combined with epinephrine for several reasons. As this patient developed hypoalbuminemia (1.9 g/dL), which increases free plasma local anesthetics concentrations and the risk of LAST, lidocaine was considered a safer option than ropivacaine due to its lower protein binding ratio. Furthermore, adding epinephrine can decrease the absorption speed and increase the maximum dose of lidocaine, as well as achieve longer analgesic duration [15]. In this regard, we selected 0.5% lidocaine combined with epinephrine.

Although dexmedetomidine could be useful, we chose a combination of ketamine and propofol in the current case. Either choice can provide analgesic and sedative effects without respiratory suppression; however, ketamine was considered more suitable to immediately treat intraoperative pain due to its rapid onset. We used propofol both for sedation and to weaken the ketamine’s adverse effects [16].

### Conclusion

In this report, we presented a case of anesthetic management with ultrasound-guided subcostal TAPB in a patient with RDEB. It is suggested that subcostal TAPB can be a useful option for peritoneal dialysis catheter placement in patients with RDEB.

## Abbreviations

LAST: Local anesthetic systemic toxicity
RDEB: Recessive dystrophic epidermolysis bullosa
TAPB: Transversus abdominis plane block
